# Syphilitic Reinfection with Wassermann Reaction Still Positive

**Published:** 1913-02

**Authors:** 


Syphilitic Reinfection with Wassermann Reaction Still Positive.—Gittings, *Brit. Med. Jour.* Two years after the first infection, during which time mercurial inunctions had been used, the patient developed a fresh lesion, containing living spirochetes, five weeks after the second exposure.

